# A Semantic Data-Based Distributed Computing Framework to Accelerate Digital Twin Services for Large-Scale Disasters

**DOI:** 10.3390/s22186749

**Published:** 2022-09-07

**Authors:** Jin-Woo Kwon, Seong-Jin Yun, Won-Tae Kim

**Affiliations:** Future Convergence Engineering Major, Department of Computer Science and Engineering, Korea University of Technology and Education, Cheonan 31253, Korea

**Keywords:** balanced partitioning, computing load prediction, digital twin, distributed computing, large-scale disasters, load balancing, machine learning, semantic data

## Abstract

As natural disasters become extensive, due to various environmental problems, such as the global warming, it is difficult for the disaster management systems to rapidly provide disaster prediction services, due to complex natural phenomena. Digital twins can effectively provide the services using high-fidelity disaster models and real-time observational data with distributed computing schemes. However, the previous schemes take little account of the correlations between environmental data of disasters, such as landscapes and weather. This causes inaccurate computing load predictions resulting in unbalanced load partitioning, which increases the prediction service times of the disaster management agencies. In this paper, we propose a novel distributed computing framework to accelerate the prediction services through semantic analyses of correlations between the environmental data. The framework combines the data into disaster semantic data to represent the initial disaster states, such as the sizes of wildfire burn scars and fuel models. With the semantic data, the framework predicts computing loads using the convolutional neural network-based algorithm, partitions the simulation model into balanced sub-models, and allocates the sub-models into distributed computing nodes. As a result, the proposal shows up to 38.5% of the prediction time decreases, compared to the previous schemes.

## 1. Introduction

As various environmental problems, such as the global warming, become severe and extreme, natural disasters become extensive and cause huge damages in recent decades [1,2,3,4]. For this reason, it is difficult for disaster management agencies to represent the complicated natural phenomena and provide rapid disaster prediction services. Digital twins can efficiently provide the prediction services by using high-fidelity models and real-time observational data [5]. However, since the models may require massive computing loads and long prediction times, the service response times can be increased, resulting in the unmanageable damages of disasters [6]. Therefore, novel acceleration schemes for the prediction services are necessary to solve this problem.

Digital twins with distributed computing schemes are applied to accelerate the prediction services by dividing the complex models into multiple sub-models and process the models on several distributed computing nodes in parallel [7,8,9,10,11]. In every simulation step, the computing nodes have to stand by until the slowest node is complete to synchronize the computation process between the nodes, which is the main delaying factor in the distributed computing schemes [12,13]. Hence, the sub-models need to contain the balanced amount of computing loads, in order to enhance the efficiency of the distributed computing, so that the waiting time between each node can be minimized [14,15]. Therefore, balancing the computing loads is the major feature for distributed computing-based prediction services.

The previous distributed computing schemes proposed diverse load balancing methods to support the time-demanding prediction services for IoT network and natural disasters [16,17,18,19,20]. Especially the methods for natural disasters estimate the computing loads with the initial number of computing points of disasters, such as the number of ignition points in wildfires, which may affect the amounts of the computing loads. However, the initial number of the computing points can be changed by correlated factors of natural disasters, such as landscapes and weather conditions, when disasters and their prediction services are in progress. Therefore, this could decrease the accuracy of the computing load prediction, cause unbalanced load distributions, and, eventually, increase the service response times with inappropriate disaster responses.

To solve the problem, we propose a novel semantic data-based distributed computing framework to accelerate the prediction services for large-scale and extensive natural disasters. The framework preprocesses the environmental data of disasters and combines them into disaster semantic data to represent the initial disaster states, such as the initial sizes of wildfire burn scars and fire fuel models. The framework performs the load balancing process by analyzing the combined environmental data. First of all, the framework accurately predicts the computing loads using an offline trained CNN (convolutional neural network) model with the disaster semantic data as the input. Second, the framework partitions the disaster semantic data into sub-models that contain a balanced amount of computing loads. Then, the framework distributes the sub-models to multiple computing nodes.

The rest of the paper is organized as follows. The related works are discussed in Section 2. The proposed framework is presented in Section 3. Section 4 shows case study-based experiments and provides result analyses. Finally, the conclusion and future works are given in Section 5.

## 2. Related Works

The main purpose of this work is the acceleration of the digital twin prediction services, in order to rapidly respond to natural disasters. For that reason, it is important to accurately predict the computing loads, based on the disaster semantic data, as well as to fairly distribute the loads. In this section, the previous studies for distributed computing methods are discussed.

As a part of the distributed computing schemes, the study of [16] proposed an edge computing-based smart city IoT system energy load prediction scheme using a machine learning algorithm. They predict the required IoT energy load on time with semantic data, including the locations and work times of IoTs. However, since the energy load prediction requires a certain amount of time for data acquisition, it is inappropriate for safety-critical events, such as natural disasters.

Guo [17] proposed a wildfire spread prediction framework, including a low-resolution wildfire spread profile-based partitioning scheme. The scheme partitions the simulation input data, based on the initial number of ignition points. Cordasco [18] proposed a distributed agent-based simulation framework to ensure the scalability and ease of a distributed environment. They assume all computing points have constant computing loads and distribute the computing loads based on the density of the points. Yue [19] proposed a framework for better computational intensity predictions and accelerations of geo-processing systems. They applied a machine learning algorithm to predict the computing loads and fairly distribute the loads based on geo-processing semantic data. Roberts [20] proposed a distributed computing scheme to accelerate the storm surge prediction. They estimate the dynamically changing load balance between partitions by the number of cells. However, since the studies only take the number of computing points into consideration, the computing load prediction can be inaccurate, causing unbalanced load distributions. For example, when a fire is spreading from one spot, the states of the fire, such as the intensity and direction, are affected by the environmental factors around it. Therefore, without considerations regarding the correlations between them, the service response times and damages are increased.

In order to overcome the limitations of the existing approaches, we propose a novel distributed computing framework with semantic analyses of correlations between the environmental data of disasters. For the analyses, the framework combines the data into disaster semantic data to represent the initial states of disasters. Based on the disaster semantic data, the framework can accurately predict the computing loads for the prediction services using a machine learning algorithm. From the prediction results, a balanced partitioning algorithm is applied to distribute the loads fairly. Since balanced amounts of computing loads can minimize the waiting time between computing nodes, the proposed framework can accelerate the digital twin prediction services. On the other hand, the past literatures only consider the number of computing points of cells to determine the computing loads.

## 3. Proposed Framework

### 3.1. Framework Overview

**Figure 1:**
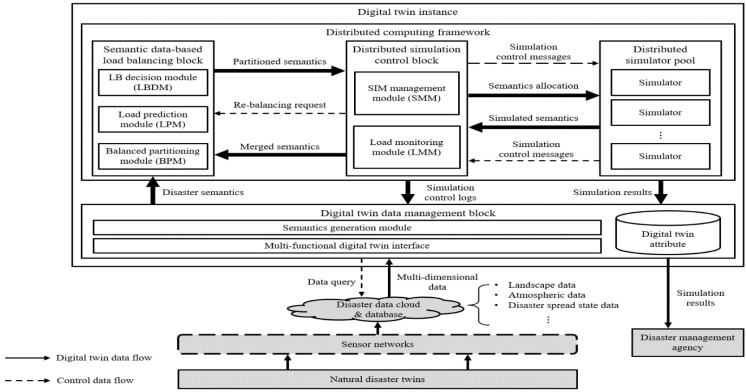
The overview of the prediction service for disasters with the proposed distributed computing framework.

The proposed framework of this paper is described in Figure 1. The framework is a functional element of a disaster digital twin instance. The framework is composed of four parts: the digital twin data management block, semantic data-based load balancing block, distributed simulation control block, and distributed simulator pool. The digital twin data management block receives multidimensional environmental data observed from the IoT sensor networks and processes them into disaster semantic data. The semantic data-based load balancing block decides the load balancing process by conducting semantic analyses of the combined environmental data, predicts the computing loads, and distributes the semantic data based on the prediction results. The distributed simulation control block manages the distributed simulators and keeps the loads balanced between the distributed semantic data. The distributed simulator pool may include multiple types of simulators that can be selected based on target applications. The simulated results are stored in the digital twin attribute of the digital twin data management block and can be used by disaster management agencies to properly respond to natural disasters.

### 3.2. Definition of the Disaster Semantic Data

**Figure 2:**
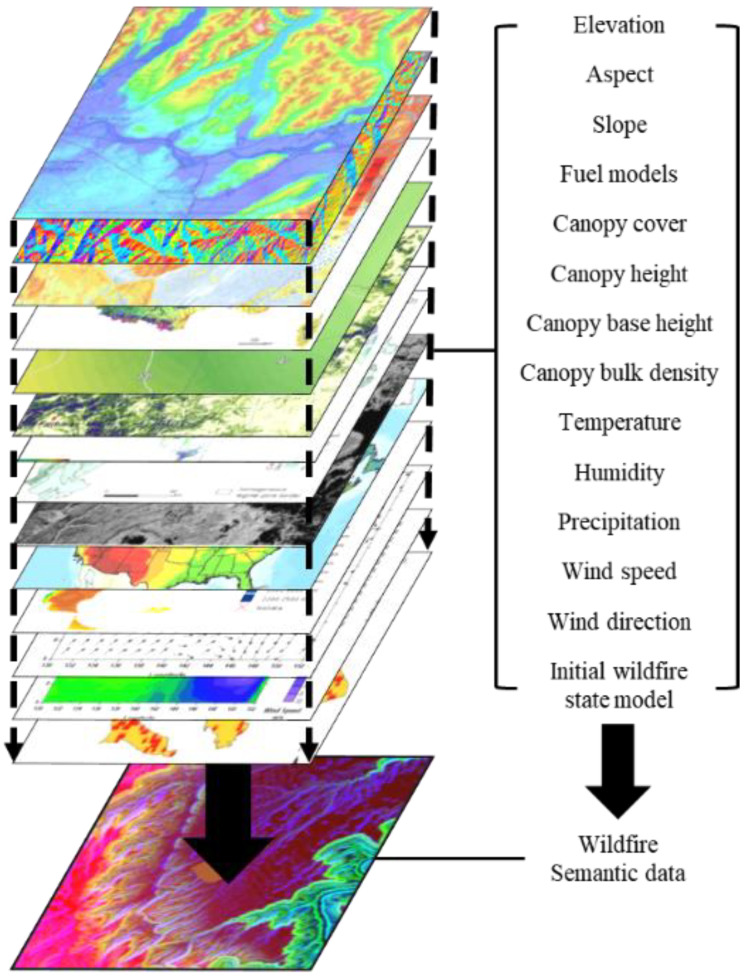
A disaster semantic data example for a wildfire.

In this paper, we define the disaster semantic data as the combination of the disaster environmental data to represent the initial states of disasters, such as the sizes of wildfire burn scars and fuel models. The sensor networks collect the environmental data from the real world’s natural disasters. The environmental data include topographic data, atmospheric data, weather data, and disaster spread data. The details of each data are described in Section 3.3. Since it is difficult to specify and describe the states of the disasters with only individual data, the correlations between the environmental data need to be considered to precisely represent the disaster states. An example of a disaster semantic data is depicted in Figure 2.

The disaster semantic data in Figure 2 consists of 14 environmental data layers. When a wildfire spreads, the fire is affected by the data around an ignition point. The 14 layers can be categorized in three parts, i.e., landscape, weather, and initial wildfire perimeter data. The landscape data include elevation, aspect, slope, fuel models, canopy cover, canopy height, canopy base height, and canopy bulk density data. The weather data contains temperature, humidity, cloud cover, precipitation, wind direction, and wind speed data. The initial wildfire state model is an initial wildfire perimeter vector data. The process of generating the semantic data is described in Section 3.3.

### 3.3. Digital Twin Data Preprocessing Module

In this subsection, a specific process of generating the disaster semantic data is described, with an example of a wildfire. When a wildfire occurs, the sensor networks including altimeters, satellite cameras, anemometers, and IR sensors collect the disaster environmental data. The data consist of landscape, weather, and initial disaster spread data and are stored in IoT databases or clouds. Landscape data refers to the geographical data, including elevations, slopes, fuel models, etc. The weather data include wind speeds and directions, temperatures, humidity, etc. The disaster spread data represents, for example, a geological array of initial ignition points where wildfire flames are growing. The preprocessing procedure is displayed in Algorithm 1.

To gather the required environmental data from the IoT database, the digital twin instance queries to the database with longitude and latitude coordinates of the location of the target disaster. Since the coordinates are based on the elliptical surface of the earth, it is necessary to apply a two-dimensional space projection method to adjust geographical errors of the collected environmental data. Then, a spatial reference on the area is set to the projected data, and the data are clipped into proper sizes for simulation inputs. The clipped data are combined as the disaster semantic data describing the states of the target disaster. The preprocessed semantic data are now sent to the semantic data-based load balancing block for the semantic analyses-based load balancing process.
**Algorithm 1** Digital twin data preprocessing$T_{td}\leftarrow \;$ type of target disaster$N_{d}\leftarrow \;$ the number of target disaster types $lat_{td},lon_{td}\leftarrow \;$ boundary coordinates of the target disaster M←  empty // Semantic data $Num_{env}\leftarrow \;$ the number of environmental data types**while**$N_{d}<0$**do**     Q← $\mathrm{Environmental}\_\mathrm{Data}\_\mathrm{Query}\left(lat_{td},lon_{td}\right)$     S← Save_to_digital_twin_data_storage     **for**
i=1 $\mathrm{to}\;Num_{env}$
**do**          $P_{i}\leftarrow$ $\mathrm{Project}\_\mathrm{environmental}\_\mathrm{data}\left(S_{i}\right)$          $P_{i_{SR}}\leftarrow$ $\mathrm{Set}\_\mathrm{Spatial}\_\mathrm{Reference}\left(P_{i}\right)$          $E_{i}\leftarrow$ $\mathrm{Calculate}\_\mathrm{Extent}\_\mathrm{to}\_\mathrm{Clip}\left(P_{i_{SR}}\right)$          $C_{i}\leftarrow$ $\mathrm{Clip}\_\mathrm{Environmental}\_\mathrm{Data}\_\mathrm{by}\_\mathrm{Extent}\left(\;P_{i_{SR}},\;E_{i}\right)$          $M_{i}\leftarrow$ $\mathrm{Merge}\_\mathrm{Clipped}\_\mathrm{Environmental}\_\mathrm{Data}\left(M_{i},\;C_{i}\right)$          **return**
$M_{i}$ // Semantic data     **end for****end while**

### 3.4. Semantic Data-Based Load Balancing Block

#### 3.4.1. Load Balancing Decision Module (LBDM)

In this subsection, a semantic analysis and the load balancing process determining operation are introduced. This module then decides which load balancing process can better improve the prediction efficiency. For example, with disaster semantic data representing a small-sized disaster, the elapsed time of a standalone or distributed simulation can be small, since the semantic data may contain a small number of computing points. Moreover, the distributed simulation time can be short, regardless of the amount of the load imbalance, since the simulation time itself is already small. Therefore, it may be unnecessary to apply the load balancing method with small-scale disasters. However, large-scale disaster semantic data require a massive standalone simulation time, since the semantic data contain a large number of computing points. For this reason, distributed computing is necessary for large-scale disaster simulations. As load imbalance can result in a huge simulation time in distributed computing, load balancing needs to be applied. Even though the load balancing process takes a certain amount of time for load prediction and balanced partitioning, the process can compensate for the standalone simulation time. The detailed simulation times of the distributed computing are described in Section 3.4.3.

Moreover, in a running simulation, the states of a disaster can be changed, and the sizes and environments of the disasters can also be changed. As the various sizes of semantic data result in different distributed computing efficiencies, it is necessary to determine the load balancing process by analyzing the disaster semantic data. According to the determined load balancing process, the semantic data of small-sized disaster semantic data are transferred to the balanced partitioning module, and the large-sized disaster semantic data are sent to the load prediction module. The detailed process of this module is described in Section 4 with a case study.

#### 3.4.2. Load Prediction Module (LPM)

In this paper, the term computing loads indicates the simulation times to predict the states of disasters, based on the disaster semantic data. The LPM uses a machine learning model to predict the simulation loads. For example, when a wildfire occurs, a fire spreads from one point to its neighboring regions, and the spot is affected by surrounding factors, including slopes, fuels aspects, weather data of winds, humidity, precipitation, and fire spreading points. In short, for the prediction process, the computing loads occur according to the semantic data around the computing points.

**Figure 3:**
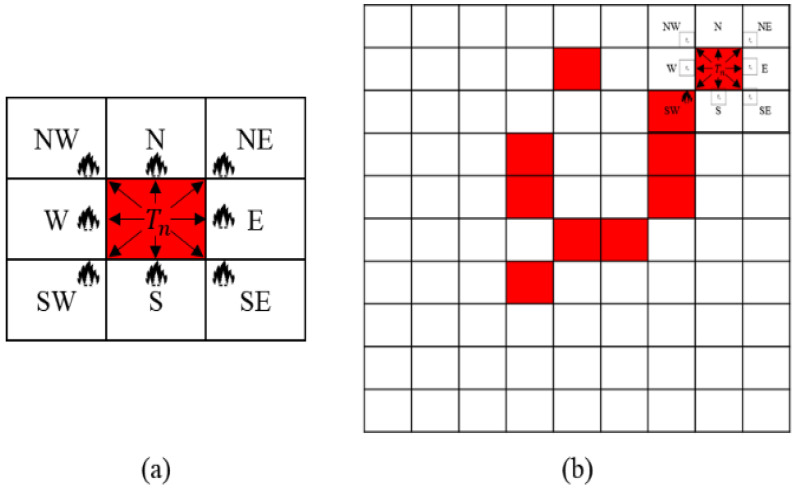
The computing points affected by the neighboring semantic data in a wildfire: (**a**) basic wildfire propagation from one ignition spot to surrounding areas; (**b**) only one spot of a wildfire is insufficient to represent the overall disaster states represented with the red spots and computing loads of a wildfire.

**Figure 4:**
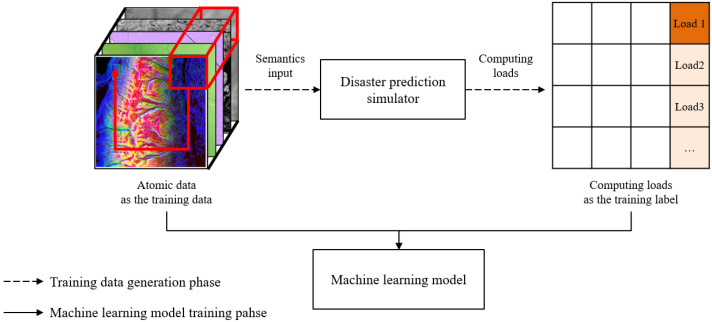
The supervised learning process.

To predict the computing loads, which is the relationship between the computing points and semantic data, a CNN (convolutional neural network) model is used specialized for drawing out the relations between neighboring pixels of images. Figure 3a displays an example of a basic wildfire spread propagation behavior on a raster data format of a wildfire simulation. When a fire spreads from a spot as a computing point in a simulation, the fire is affected by the surrounding semantic data. However, Figure 3b shows that it is difficult to comprehend the overall states and computing loads of the disaster with only the area of Figure 3a. This is because when a wildfire spreads, the fire grows with various behaviors and can spread to distant spots, which are called spot fires. To solve the problem, setting a certain size of the semantic data is necessary to represent the basic wildfire propagation areas for predicting the computing loads. The standard size of the semantic data is defined as the atomic data. The details of the atomic data are discussed in Section 4. Figure 4 depicts the supervised learning process to train the correlations between the atomic data and computing loads. The atomic data are the inputs of disaster prediction simulators, and the simulation outputs are the computing loads corresponding to the inputs. As a result, the machine learning model is trained with the atomic data as training data and computing loads as output data or labels.

**Figure 5:**
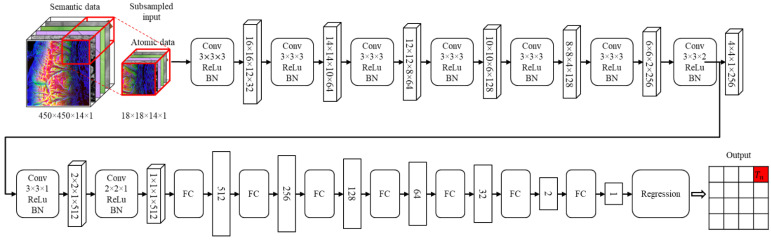
An example of the CNN-based machine learning model for the computing load prediction.

An example of the machine learning model configuration for the computing load prediction is described in Figure 5. The atomic data are subsampled from the semantic data and sequentially computed through the computing load prediction process. The process includes computations through convolutional layers, activation functions, batch normalization (BN) layers, and fully connected layers, with the linear regression function as the very last layer of the model. Since the regression function predicts the computing load outputs from given input atomic data, the mean squared error (MSE) loss function is applied as the model performance metric. The configurations of the model can be different, depending on the characteristics of the semantic data. As a result, the LPM makes a load map with the predicted computing loads of every atomic data, and the load map is sent to the balanced partitioning module.

#### 3.4.3. Balanced Partitioning Module (BPM)

**Figure 6:**
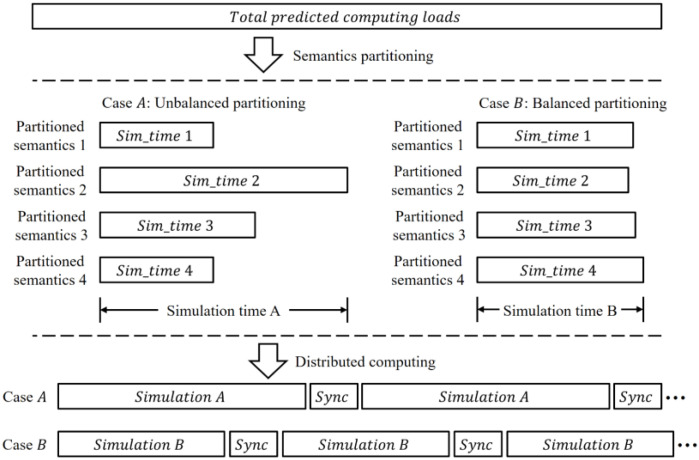
The simulation time comparison, in terms of balanced computing loads.

The reason for the partitioning and distributed computing is depicted in Figure 6. To run another distributed simulation step after one step, every partitioned simulation session needs to be complete. Figure 6 shows the two cases of partitioning, i.e., unbalanced and balanced partitioning. In the case of A, each partitioned semantic data requires a different simulation time. In particular, since the partitioned semantic data 2 requires more simulation time than the others, the other simulation sessions have to wait until the simulation for partitioned semantic data 2 is complete, which is described as simulation time A.

Nevertheless, in case of B, as the simulation times are nearly alike, the overall waiting time between the nodes is smaller than the waiting time of case A, and the overall simulation time is shorter than the simulation time of case A. Moreover, given the same amount of time to both cases, the balanced partitioned case of B can run even more simulation steps than case A. In this paper, the synchronization delays are assumed to be constant. Therefore, the computing loads of the partitioned semantic data have to be balanced, in order to accelerate the simulation processes, by following equation 1:

Load 1 ≈ Load 2 ≈ Load 3 ≈ Load 4(1)

**Figure 7:**
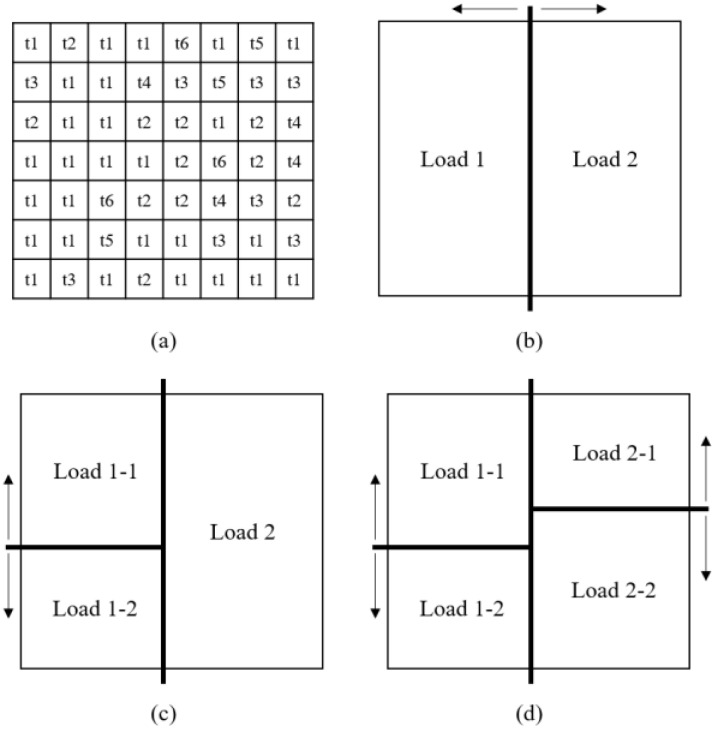
The balanced partitioning process, based on the k-d tree algorithm: (**a**) predicted computing load map of disaster semantic data; (**b**) dividing the load map based on the x-axis into balanced Loads 1 and 2; (**c**) dividing the Load 1 into the two balanced loads based on the y-axis, Loads 1-1 and 1-2; (**d**) partitioning Load 2 into Load 2-1 and Load 2-2, following the y-axis.

The balanced partitioning process is illustrated in Figure 7, and the partitioning algorithm is shown in Algorithm 2. The partitioning process follows the k-d tree algorithm, since the algorithm searches the distribution points and equally divides the models through the binary searching method on each partition.
**Algorithm 2** Balanced partitioning based on k-d tree algorithmCapability←  required computing power of one distributed computing nodeLoad←  computing load of each partitionAxis←  partitioning base axis of semantic dataCnt←  partitioning loop countCnt=2Axis=XLoad← Balanced_partitioning(Load,Axis)**while**Load>Capability **do**     **if**
Axis=X **then**          Axis=Y     **else if**          **then**
Axis=X     **end if**     **for**
i=0 to Cnt **do**          Load←Balanced_partitioning(Load, Axis)     **end for**     Cnt=Cnt×2**return**Load // balanced loads between partitioned models**end while**

### 3.5. Distributed Simulation Control Block

**Figure 8:**
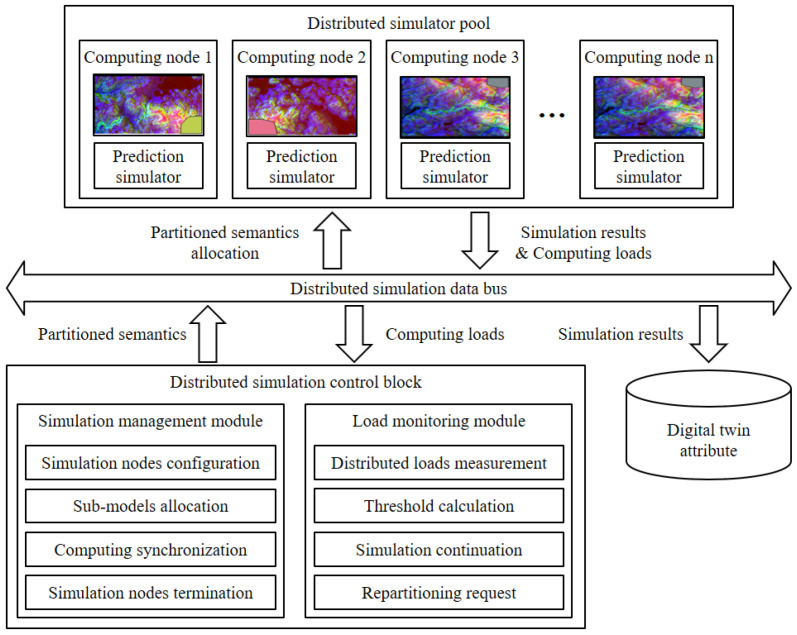
The simulation control block manages the distributed computing procedure.

In this subsection, the distributed simulation control block is introduced. The operation of the block is illustrated in Figure 8. The control block allocates the partitioned semantic data on the distributed computing nodes and controls the simulation sessions. The simulation results are transferred to the load monitoring module or to the digital twin attribute in the digital twin data management block.

#### 3.5.1. Simulation Management Module (SMM)

The partitioned semantic data from the balanced partitioning module are sent to the simulation management module. The module configures the distributed computing nodes to simulate the partitioned semantic data. The module allocates the semantic data to each distributed computing node. At the end of each simulation step, the module synchronizes the simulation results of the nodes. In every simulation step, each computing node sends a simulation result to the digital twin attribute and transfers the computing loads of the nodes to the load monitoring module. When all the simulation steps are finished or there are any simulation interrupts, the management module terminates the computing nodes and sends the results to the load monitoring module or digital twin attribute.

#### 3.5.2. Load Monitoring Module (LMM)

The load monitoring module examines the loads of the nodes to continuously balance the computing loads to satisfy Equation (1) in Section 3.4.3. In case the module detects the load imbalance, the module merges the distributed disaster semantic data of the current simulation time and sends the semantic data to the load balancing block with repartitioning request messages. From the load balancing block, the overall procedure of the computing load prediction, balanced partitioning, and distributed computing are executed for the merged semantic data. The computing load balance between the simulated semantic data is decided based on Equations (2)–(4), as follows:

(2) $$T=CL_{max}-CL_{min}\;$$

(3) $$T^{\mathrm{Threshold}}=\left(\frac{1}{m}\sum_{a=1}^{m}CL^{a}\right)*10\%\;$$

(4) $$T\geq T^{\mathrm{Threshold}}$$

$CL_{max}$ means the longest simulation time or the largest computing load, and $CL_{min}$ denotes the shortest simulation time. The amount of the load imbalance T is determined by the difference between $CL_{max}$ and $CL_{min}$. $T^{\mathrm{Threshold}}$ means the threshold of the load imbalance and can be calculated as the 10% of the average computing loads, where m is the number of partitions or computing nodes. If T of Equation (2) is equal to or larger than the threshold like Equation (4), LMM determines that the computing loads are unbalanced, and the repartitioning process is conducted.

### 3.6. The Data Flow of the Digital Twin Service for Disaster Predictions

**Figure 9:**
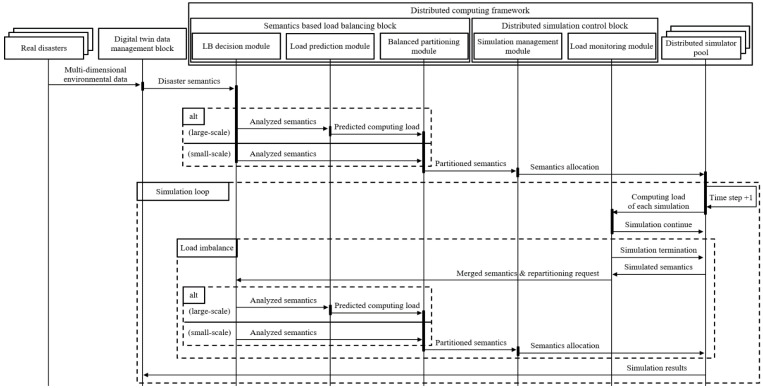
The sequence diagram of the digital twin service for large-scale disasters.

The overall procedure of the digital twin service for large-scale disasters using the proposed distributed computing framework is illustrated as a sequence diagram in Figure 9. The sensor networks collect the IoT sensor data, including disaster environmental data, from the target disaster areas. The data are stored in the database and data clouds. The digital twin instance queries to the database and stores the multi-dimensional data as digital twin data in the digital twin data management block. The digital twin data are preprocessed as disaster semantic data to represent the initial states of a target disaster. The semantic analyses for the merged data are conducted to determine the load balancing process, for example, analyzing the size of a disaster. According to the analyses, the semantic data are sent to the computing LPM or directly to the balanced partitioning module. The partitioned semantic data are transferred to the simulation management module in the distributed computing control block. The module configures the distributed computing nodes and allocates the partitioned semantic data. The nodes’ computing loads are continuously monitored in every simulation step by the load monitoring module. The simulations continue until the load imbalance or simulation interrupts are detected. When the module detects the load imbalance, based on the threshold from Equations (2)–(4), the simulation management module terminates the nodes, and the semantic data are merged and sent to the load balancing block with repartitioning request messages. From the load balancing block, the overall procedure is repeated. The results of the simulations are stored in the digital twin attribute and can be used by disaster management agencies.

## 4. Experiments and Analysis

In this section, a case study of a disaster is described. It is mainly based on a wildfire spread prediction simulation. The experimental setup and specifications are shown in the following subsections. Additionally, the experimental results are discussed in the Section 4.5.1 and Section 4.5.2.

### 4.1. Simulation Description

In this work, a case study was applied to validate the performance of the proposed framework. The case study was based on a wildfire spread prediction simulator, FARSITE, developed by the US Department of Agriculture (USDA) [21]. FARSITE contains wildfire propagation algorithms, such as Huygens’ principles and Rothermel equations [22]. The algorithms include various types of the wildfire propagation behaviors, including surface, spot, and crown fires. The inputs of the simulator are the wildfire environmental data, including the landscapes, weather data, and initial wildfire state models of the wildfire burn scars. Based on the cellular and raster format of the input data, the simulator also shows the characteristics of wildfire behaviors, such as the rate of spread, wildfire intensities, and wildfire spreading shapes.

### 4.2. Distributed Computing Configuration

**Figure 10:**
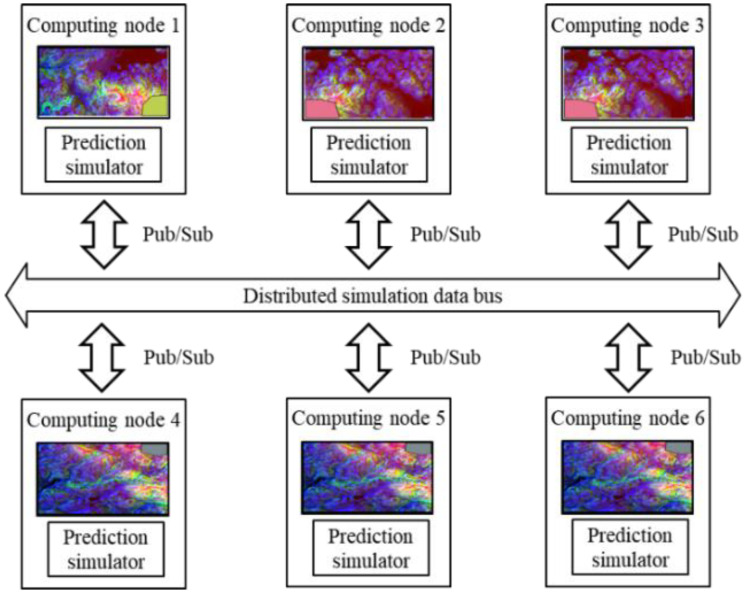
The architecture of the distributed computing scheme.

The proposed framework was implemented on the authors’ laboratory desktop PC. The PC was equipped with a 10-core CPU, 20-core memory, and 3.70 GHz Intel(R) core (TM) i9-10900KF CPU processor. For the machine learning training, a 10.0 G GPU NVIDIA GeForce RTX 3080 device was used. The distributed computing followed the environment of the PC. The distributed computing nodes were connected through the pub/sub-based middleware data bus and allocated the same computing powers. In this paper, the communication overheads for pub/sub and synchronization between the nodes were not considered. The architecture of the distributed environment is shown in Figure 10.

### 4.3. Dataset Description

**Figure 11:**
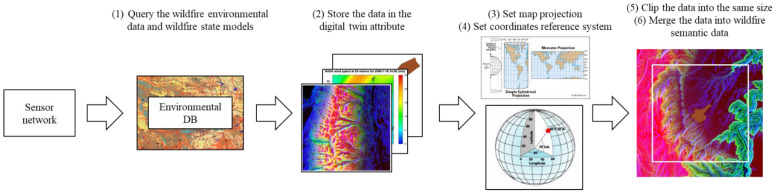
The wildfire digital twin data preprocessing procedure.

Through the semantic data generation module in Section 3, the semantic data were generated by combining the environmental data of landscapes, weather data, and initial wildfire state models. This subsection demonstrates the way the disaster semantic data were created, with an example of a wildfire. The preprocessing procedure was displayed in Figure 11.

The wildfire landscape data can be acquired by calculating the longitude and latitude of the target wildfire spot and querying to the environmental database. The data were stored in the digital twin attribute. Since the longitude and latitude are calculated on the elliptical surface of the Earth, it is necessary to project the data into a 2D map to set the input for the simulation. In this study, the map projection method was based on the commonly used Mercator projection, with the coordinates reference system of EPSG:4326. To simulate a wildfire with the projected data, the whole of the environmental data needed to be clipped to the same size. The weather data, such as temperature, humidity, wind direction, and wind speed, were set to be random constants. The initial wildfire state models were also queried to the database. Finally, the semantic data can be created by merging the clipped environmental data and initial wildfire state model.

In this study, the experimental data of the wildfire were based on the initial states of real wildfires in the US in 2016. The real landscape data of 2016 wildfire scenarios can be collected from the USDA LANDFIRE database [23]. The weather data were customized with random constants bound to the default value ranges of FARSITE. The initial wildfire state models were from the database of the National Interagency Fire Center (NIFC) [24]. The scenarios were randomly selected from the large-scale wildfires. The standard of the wildfire sizes followed the definition of the USDA fire terminology and NWCG (national wildfire coordinating group) [25,26].

The semantic data for the experiments followed the raster format of 450 × 450 pixels. The length of each pixel represents 30 m distance in the real world. Moreover, according to the fire terminology from the USDA, large-scale wildfires can spread more than 300 acres. Therefore, to cover the area of large-scale wildfires, the semantic data sizes were set up to 450 × 450 pixels raster data.

### 4.4. Experimental Scenarios

The experimental data were composed of the training, test, and validation data for a CNN-based machine learning model. For model training, 51 cases of randomly selected large-scale wildfires were used. Among the 341 wildfire cases, 17 wildfire cases, classified as class F and defined in [25,26], were set as the test data. The label data were the computational times for each cell of wildfire semantic data in FARSITE simulations. The CNN model was trained with the semantic data as the input and computing loads as the output. The sizes of the atomic data ranged from 9 × 9 to 21 × 21, and the number of partitions ranged from 2 to 32. The numbers of partitions and computing nodes were squared numbers of 2, as the partitioning algorithm followed the k-d tree.

### 4.5. Experimental Results and Analysis

In this section, the proposed framework is evaluated, in terms of the relative simulation times for wildfire spread prediction using FARSITE. The experiments were conducted between the standalone simulation and previous distributed computing methods, uniform-sized, and points-based load balancing schemes. The uniform-sized scheme means that the semantic data are partitioned into the exact same size, no matter how many computing points a partition can contain. The point-based scheme describes that the load balancing process is only based on the number or the density of the computing points. The relative simulation time of each method can be calculated by dividing the maximum computing load of each distributed computing scheme with the load of the standalone simulation. The equation is described as follows:

(5) $$T^{Scheme}=\sum \frac{\max\left(CL^{Scheme}\right)}{CL^{Standalone}}/f\;$$

$T^{Scheme}$ means the average relative simulation time of each distributed computing scheme. The value $\max\left(CL^{Scheme}\right)\;$ is the maximum value the computing loads of each distributed computing scheme, and $CL^{standalone}$ denotes the computing load of the non-distributed method. The denominator f means the number of wildfires used for the experiments.

The relative simulation times for the proposal are discussed based on two load balancing parameters, i.e., the atomic data size and number of partitions. The simulation time for each number of partitions and atomic data size a can be calculated as the average of the computing loads and the computing load of the standalone simulation. The average is divided by the number of wildfire scenarios. The evaluating equation is described in Equation (6) as follows:

(6) $$t_{a}^{ML_{DC}}=\sum \frac{\max\left(CL_{a}^{ML}\right)}{CL^{Standalone}}/f\;$$

$t_{a}^{ML_{DC}}$ means the relative simulation time of each distributed computing scheme, based on the atomic data size of a. The numerator max $\left(CL_{a}^{ML_{DC}}\right)$ indicates the maximum computing load of machine learning-based distributed computing scheme with the atomic data size a.

#### 4.5.1. Experimental Results on Relative Simulation Time

**Figure 12:**
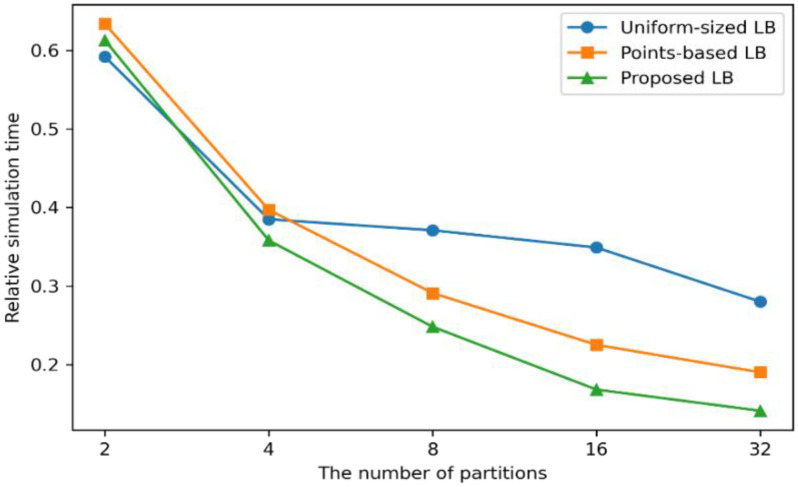
The experimental results by the number of partitions.

The main purpose of this experiment was to compare the simulation times between the standalone simulation and distributed computing methods. Figure 12 displays the relative simulation times of each method, compared to the standalone simulation with different numbers of partitions. The proposed method was based on a 21 × 21 atomic data size, since the atomic data size shows the best reduced simulation time among other atomic data sizes. The comparison between the different atomic data sizes is discussed in Section 4.5.2.

The simulation time of the uniform-sized method increases until four partitions, and the extent of the increase slows down until 16 partitions. Since the uniform-sized LB (load balancing) method divides the semantic data into the same size, the partitioning axes of 4, 8, and 16 partitions divide the semantic data with empty wildfire spreading areas. The methods showed the shortest simulation times with 32 partitions. This means that partitioning the semantic data up to 32 can barely make a partition small enough to contain the wildfire computing points. In the points-based and proposed LB scheme, the growths of the performances decreased as the number of partitions increased, especially with 16 partitions. This is because the area of semantic data in a partition gets smaller than atomic data with increases of the number of partitions. Since the atomic data refers to the minimum, but essential, amounts of semantic data to represent the computing loads, when the atomic data are divided smaller than the original size, the partitions may contain different amounts of computing loads, which can cause the simulation delays. The proposal shows approximately 52% and 25% faster simulation times than the uniform-sized and points-based method in 16 partitions, with 30% and 26% in 32 partitions. This means that the proposed machine learning model can represent more accurate computing loads than the other methods. Therefore, the proposal can support the fastest wildfire spread predictions among them.

#### 4.5.2. Experimental Results on the Atomic Data and the Partitions

**Figure 13:**
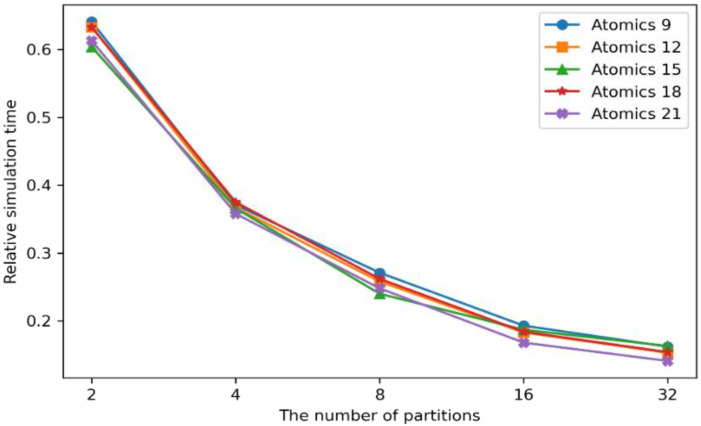
The experimental results by the atomic data sizes and the number of partitions.

Since the proposal shows the shortest simulation time with a 21 × 21 atomic data size, this experiment targeted the performances of the other atomic data sizes and numbers of partitions. Figure 13 shows the simulation time comparison between the combination of the atomic data sizes and number of partitions.

The wildfire spread algorithm basically required a 3 × 3 semantic grid. Considering the various wildfire behaviors, such as spot fires, which cause ignitions outside of the main fire areas with flying sparks and embers, the atomic data sizes ranged from 9 × 9 to 21 × 21 semantic data grids to cover the unexpected distant fires. As shown in Figure 13, the 9 × 9 atomic data size had the longest simulation time, whereas the 21 × 21-sized atomic data took the shortest time in almost every case of the partitions. This is because the machine learning model can predict the computing loads more accurately, with the more referenced semantic areas, especially for large-scale wildfires. Correspondingly, the computing loads can be balanced on the partitioned semantic data. However, the performance gaps between the different sizes of the atomic data were minor. Since it is difficult for a wildfire to spread more than 21 × 21 semantic data in normal situations, the atomic data sizes have little effect on the simulation times.

The wildfire spread simulation time can be increased with larger atomic data sizes. Since the sizes of the machine learning models are based on the atomic data sizes, the larger atomic data can make more training data set and cause higher computational resources. This also increases the computing load prediction time, which can delay the wildfire spread simulations. Moreover, huge communication overheads can be generated between the distributed computing nodes with more than 32 partitions. This is another factor that may slow down the wildfire spread simulations. Therefore, the atomic data sizes of 21 × 21 can produce the fastest wildfire spread prediction performances, with the number of partitions between 16 and 32, as discussed in the previous experiment.

## 5. Conclusions

This paper proposes a novel distributed computing framework that analyzes disaster digital twin semantic data using the CNN-based machine learning algorithm and balanced partitioning to improve digital twin services for large-scale disasters. First, the digital twin instance collects disaster environmental data from the IoT database; then, it preprocesses the data multichannel data and combines them into the disaster semantic data. Second, the load balancing process is decided by the semantic analyses of the merged data. Third, a CNN-based machine learning model is proposed to predict the computing loads from the semantic data. The machine learning model is trained by disaster scenarios with the supervised learning method to accurately feature the relation between the semantic data and computing loads. Finally, the semantic data are partitioned based on the k-d tree algorithm and distributed on the multiple computing nodes. This enables the digital twin to rapidly predict natural disasters.

For the experiments, a wildfire spread simulator FARSITE was used as a case study, with the real historic large-scale wildfires in the USA in 2016. The experimental results show that distributed computing methods can reduce the simulation time more than the standalone systems. Moreover, the proposed method obtains the shortest simulation time, compared to the other methods, with the uniform-sized and points-based load balancing method. This means the semantic analyses of the correlated environmental data of disasters can predict the computing loads accurately, so that, when the disaster semantic data are partitioned, the divided semantic data contain nearly equal amounts of the computing loads. With that, the waiting time of the distributed computing can be reduced and digital twin service response times are minimized. The amount of the reduced simulation time depends on the two load balancing parameters of this paper, sizes of atomic data, and number of partitions. In conclusion, the proposed method can accurately predict the computing loads of large-scale disasters and rapidly predict disaster states with fairly balanced loads for digital twin services.

In this paper, only the wildfire cases were considered as the experimental scenarios. Since the types of natural disasters and their models are different per system, it is necessary to develop a more generalized load balancing method. Moreover, the proposed method needs further updates to be applied to other emerging domains, such as industrial manufacturing digital twins, for the upcoming metaverse era.

## Data Availability

Not applicable.
